# Case Report: Solitary metastasis to the appendix after curative treatment of HCC

**DOI:** 10.3389/fsurg.2023.1081326

**Published:** 2023-03-30

**Authors:** Zun-Yi Zhang, Yu-Wei Wang, Wei Zhang, Bi-Xiang Zhang

**Affiliations:** Research Laboratory and Hepatic Surgery Center, Department of Hepatic Surgery, Tongji Hospital, Tongji Medical College, Huazhong University of Science and Technology, Wuhan, China

**Keywords:** HCC, metastasis, appendix, resection, systemic treatment

## Abstract

**Background:**

Liver cancer is now the fourth most common cancer in China. The most important factor in decreasing the overall survival is recurrence. Nearly 40%–70% of patients would be detected with intrahepatic or extrahepatic recurrence in 5 years after R0 resection. The intestine is not a usual site for extrahepatic metastasis. Only one case of hepatocellular carcinoma (HCC) metastasis to the appendix has been reported so far. So, it poses a difficulty for us to develop treatment plan.

**Case presentation:**

Here, we report a very rare case of a recurrent HCC patient. R0 resection was first performed on this 52-year-old men who was diagnosed with Barcelona Clinic Liver Cancer stage A HCC. Different from other cases, a solitary metastasis to the appendix was detected 5 years after the R0 resection. After discussing with the multidisciplinary team, we decided to perform surgical resection again. The final postoperative pathology confirmed HCC. Complete responses were detected in this patient after the combined treatment of transarterial chemoembolization, angiogenesis inhibitors, and immune checkpoint inhibitors.

**Conclusion:**

Because solitary metastasis to the appendix in HCC is very rare, this case might be the first reported in HCC patients after R0 resection. This case report highlights the efficacy of the combination of surgery, local regional therapy, angiogenesis inhibitors, and immune treatment in HCC patients with solitary metastasis to the appendix.

## Introduction

Liver cancer is now the fourth most common cancer in China (1, 2). Hepatocellular carcinoma (HCC) represents the majority of primary liver cancer. Nearly 40%–70% of patients would be detected with intrahepatic or extrahepatic recurrence in 5 years after R0 resection (3). The most common recurrence pattern is intrahepatic recurrence. Extrahepatic metastasis is relatively low in incidence. The most common sites of extrahepatic metastasis are the lungs, bones, lymph nodes, and adrenal glands (4). The intestine is not a usual site for extrahepatic metastasis. So far, only one case of HCC metastasis to the appendix has been reported (5). Also, the appendix was found with metastasis because the tumor lesion in the liver ruptured. Solitary metastasis after R0 resection of HCC might be the first reported after we reviewed the domestic and international literature.

Because this kind of metastasis is rare and lacks imaging features, it is easily misdiagnosed as appendicitis. We need to solve the problem of improving the accuracy of the diagnosis and prolonging the patient's survival time. According to the conventional view, extrahepatic metastasis of HCC usually means a worse prognosis (6). Most advanced-stage HCC patients would die of liver failure because of the progression of intrahepatic lesions rather than extrahepatic metastasis (7). Although with a high risk of recurrence, the selected patients with resectable extrahepatic metastasis could achieve an acceptable prognosis after R0 resection. With the improvement of medicine in liver cancer, the evolving role of immune checkpoint inhibitors (ICIs), angiogenesis inhibitors, and local regional treatment offers great promise in treating HCC patients with a high risk of recurrence (7). Nonetheless, no well-designed large samples of clinical control study have been reported. Thus, this poses a difficult problem for clinicians.

Herein, we report one case of an HCC patient with solitary metastasis to the appendix 5 years after R0 resection of the intrahepatic lesion. In this case report, we have two objectives. The first is to highlight that the appendix might be a site of tumor recurrence after R0 resection of the primary site of the liver. The second is to highlight that resection of the solitary metastasis to the appendix combined with local regional treatment, immune checkpoint inhibitors, and angiogenesis inhibitors could prolong the survival time of such patients. This study was reported in agreement with the principles of the CARE guidelines (8).

## Case presentation

**Figure 1:**
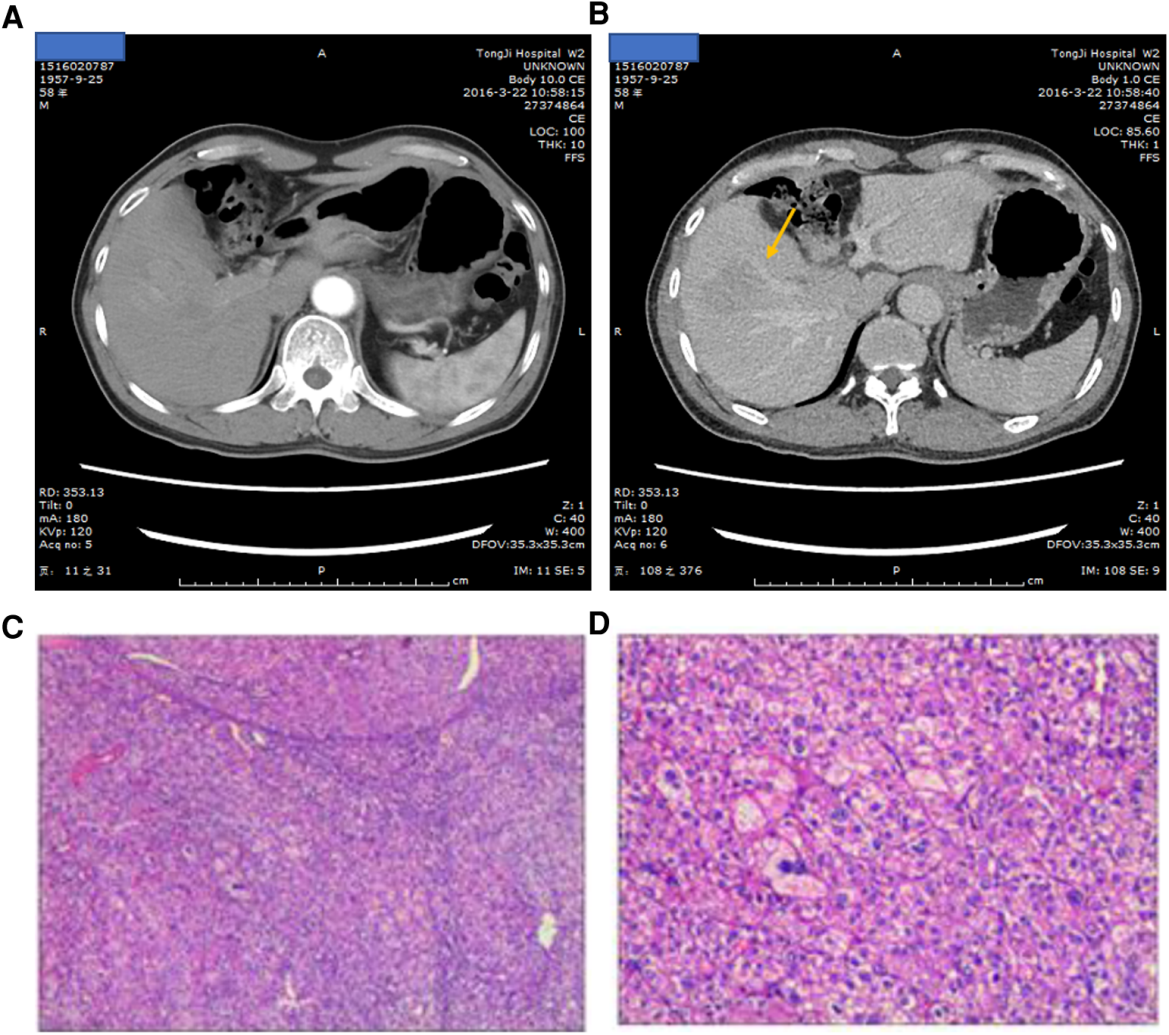
(**A**) Arterial phase of the liver-enhanced CT scan showing the tumor lesion in segments 5 and 6. (**B**) Portal venous phase of the liver-enhanced CT scan showing the tumor lesion in segments 5 and 6; the arrow points at the liver lesion. (**C,D**) HE staining of pathological diagnosis.

**Figure 2:**
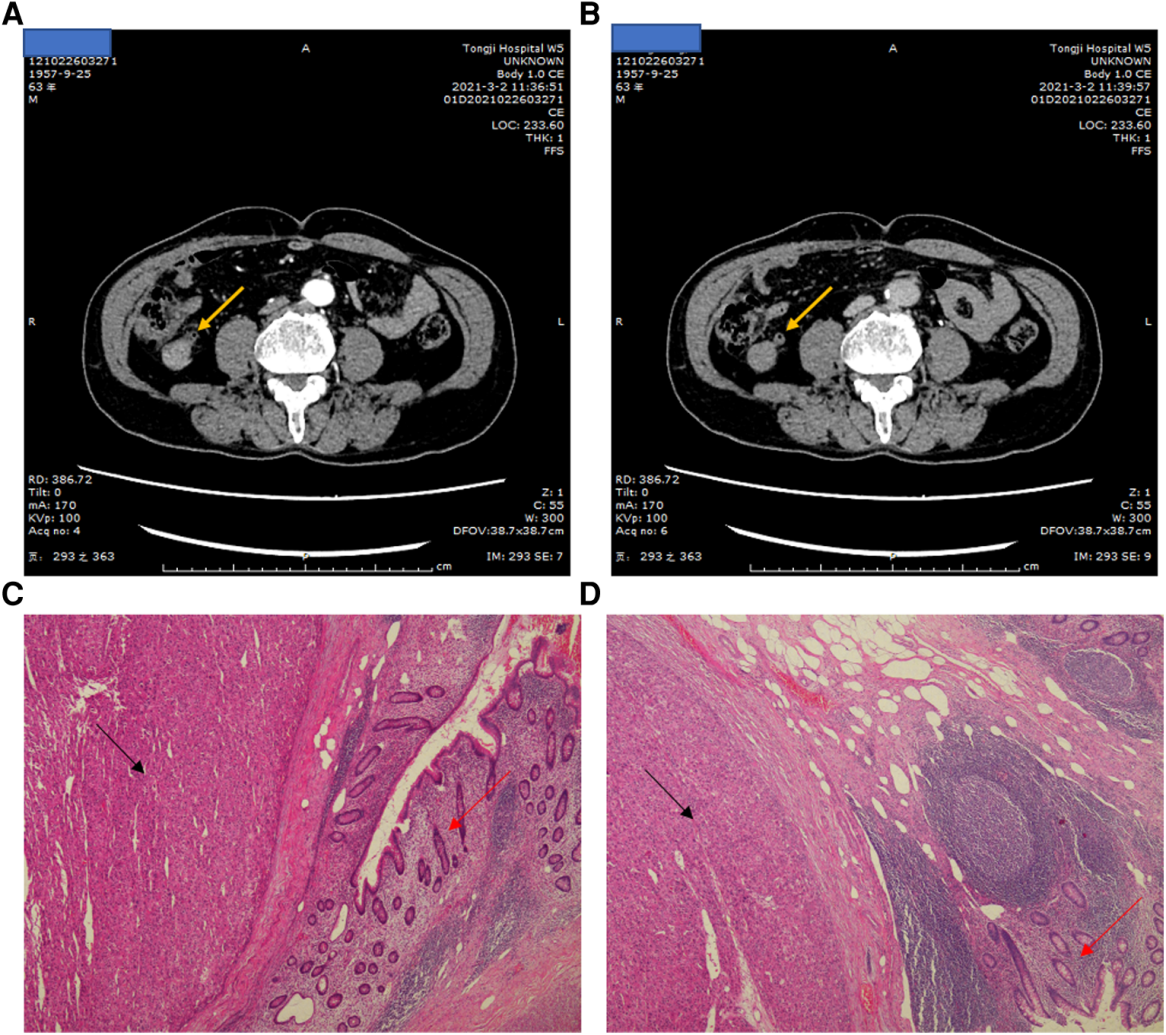
(**A**) Arterial phase of the Liver-enhanced computed tomography (CT) showing the tumor lesion in the appendix, the arrow point at the appendix. (**B**) portal venous phase of the the Liver-enhanced computed tomography (CT) showing the tumor lesion in the appendix, the arrow point at the appendix. (**C**, **D**) The HE stain of pathological diagnosis. The black arrow point at tumor lesion. The red arrow point at the appendix.

**Figure 3:**
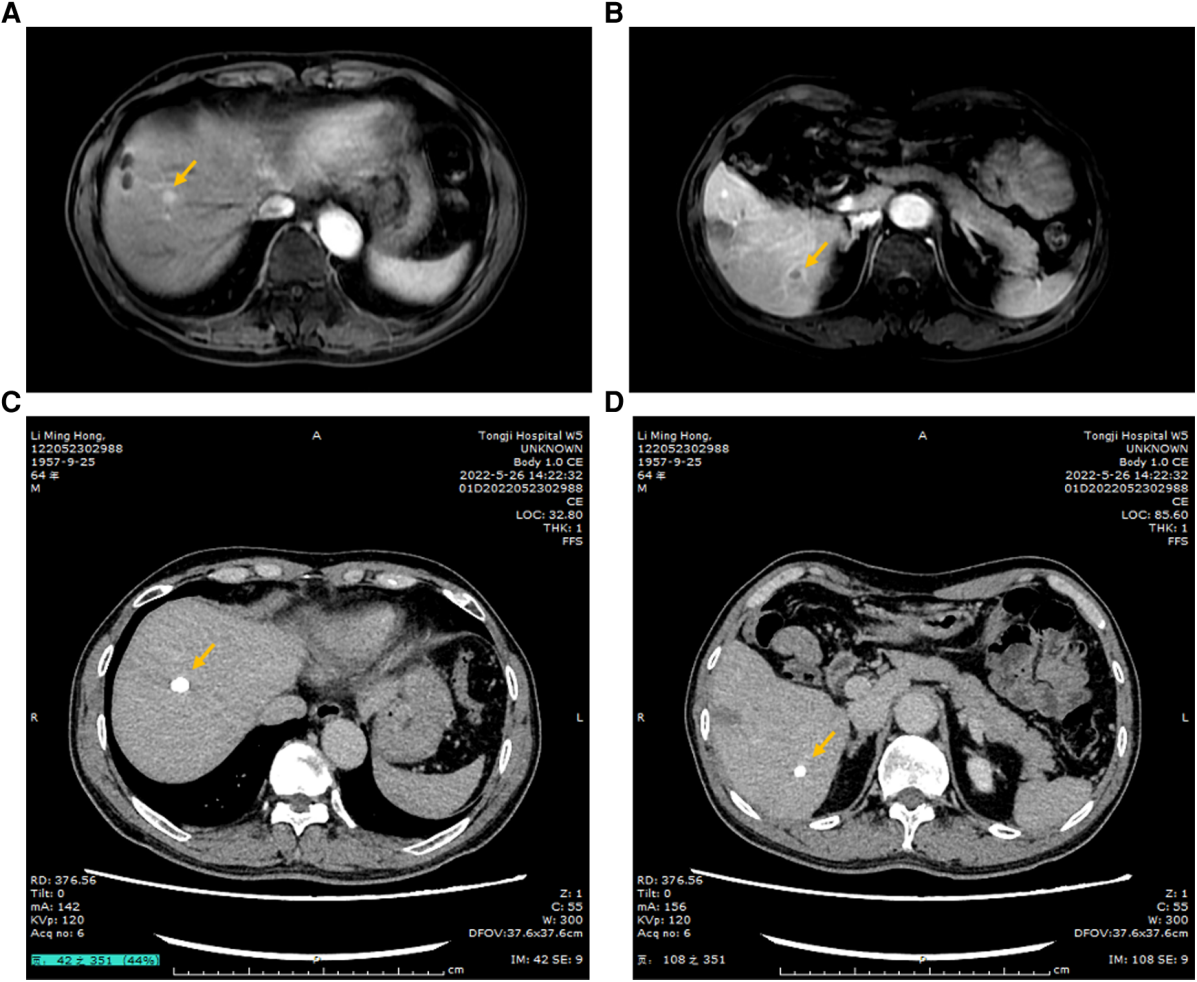
(**A,B**) PWI of the MRI scan showing the typical metastatic tumor in the liver; the arrow points at the lesion. (**C,D**) After TACE combined with angiogenesis inhibitors and ICIs, the typical lesions of the liver were covered by iodized oil, which were shown in the CT scan.

**Figure 4:**
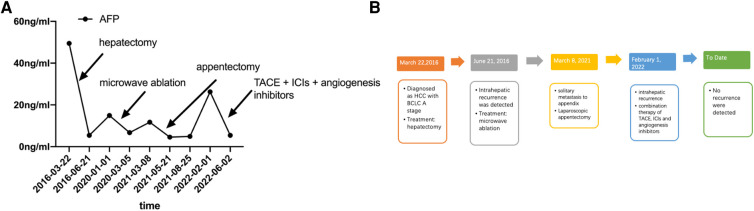
(**A**) AFP level of this patient from the first detection of HCC to the last treatment. (**B**) Timeline of the treatment for this patient.

A 52-year-old men with hepatitis B virus-associated chronic hepatitis was diagnosed with HCC at the clinic in 2016. No other specific family and psychosocial history including relevant genetic information should be reported. Physical examination showed no positive results. The patient's alpha-fetoprotein (AFP) was 49.36 ng/mL. A computed tomography (CT) scan showed that liver tumors were located in segments 5 and 6 (Figures 1A,B). The Child–Pugh Score was A with five points. The BCLC stage for this patient was A. After confirmation of no surgical contraindications, robotic-assisted laparoscopic segmentectomy 5–6 was performed on this patient. The surgery went without a hitch. The resected specimen was extracted from the lower abdomen with a specimen bag. The pathologic result confirmed the hepatic lesion was primary middle differentiated HCC (Figures 1C,D). After the resection, this patient accepted the antiviral treatment. Every 3 months, this patient came back to our department to recheck his tumor biomarkers (AFP, abnormal prothrombin DCP) and to undergo radiology tomography (ultrasonography, MRI, or CT). There was no sign of recurrence until 3 years after the surgery. On 29 December 2019, this patient returned to our department because of the elevated level of AFP. After an MRI scan, a new tumor lesion with a diameter of 2 cm was detected in segment 7. Then, microwave ablation was performed on this hepatic lesion. The AFP level soon came back to the normal range after the surgery. On 6 March 2021, this patient returned to our department for the symptoms of acute appendicitis. The AFP and DCP levels were found to be elevated slightly. After the enhanced CT scan, a tumor with a diameter of 2 cm was found to adhere to the tip of the appendix (Figures 2A,B). No sign of recurrence was detected in the remnant liver at the same time. Although it is very rare, this patient was diagnosed with solitary metastasis of HCC to the appendix. After discussing with the multidisciplinary team, laparoscopic appendectomy was performed on this patient on 12 March 2021. The pathologic diagnosis confirmed that the lesion adhered to the appendix was a metastatic HCC (Figures 2C,D). The metastatic lesion had invaded all the layers from the serous membrane submucosa. After appendectomy, this patient followed the routine follow-up every 3 months in our clinic. On 15 January 2022, this patient was found to have elevated AFP and DCP levels. After the MRI scan, we found multiple recurrent intrahepatic lesions in the right half of the liver. The maximum diameter of the recurrent intrahepatic lesion was 2 cm (Figures 3A,B). After multidisciplinary team discussion, transarterial chemoembolization (TACE) combined with angiogenesis inhibitors (lenvatinib) and ICIs (PD-1 antibodies) was administrated to this patient. After 4 months, the CT scan showed that the typical site of liver metastasis had been covered by iodized oil (Figures 3C,D). The AFP level was found to decrease to the normal range (Figure 4A). So far, this patient has survived for 7 years, and the disease was found to be in a stable state. The timeline for this patient’s treatment is summarized in Figure 4B.

## Discussion

Based on the latest report from China (9), HCC is now the fourth-most malignancy and the third leading cause of mortality. Surgical treatment including hepatectomy and liver transplantation as the only way to cure the disease is the most commonly used treatment to improve the survival of patients. However, postoperative recurrence within 5 years still could be observed in 40%–70% of patients (7). The most common sites of extrahepatic recurrence are the lungs, bone, lymph nodes, adrenal glands, and brain, in that order (4). Intestinal metastasis is not a common site of metastasis, which was reported in 0.5%–4% of HCC patients (10, 11). So far, there is only one case that has reported a patient with tumor lesions in the liver and appendix concurrently (5). The cause of metastasis to the appendix was thought to be the rupture of an exophytic HCC into the peritoneal cavity and subsequent implantation of the tumor nodule onto the serosal surface of the bowel (5). Compared with metastatis to the appendix, metastatis to the colon was relatively common. The cause of metastasis to the colon could be stratified into two major reasons. One is the implantation of tumor cells. The other one is hematogenous metastasis (5, 12). Table 1 shows a summary of patients with colon/appendix metastasis from HCC obtained after research in the domestic and international literature. The median time from initial HCC to colon/appendix metastasis is 7 years (Table 1). Among the reported cases, only two cases had a history of tumor rupture (5, 13). Three patients had metastasis to the colon/appendix without a history of tumor rupture or hepatectomy (14–16). So, the cause of the metastasis to the colon or appendix may be complex. Implantation or hematogenous metastasis could both be a major cause. Our patient reported here was found to have appendix metastatis 5 years after when the tumor was R0-resected. We thought that the reason for metastasis for this patient was implantation. During the first surgery, the HCC specimen was taken out of the patient by a specimen bag from the lower abdomen. Although we were careful during the process, cell exfoliation still could happen. After we rechecked the pathological figure, we finally confirmed that tumor tissues had invaded all the layers from the serous membrane to the submucosa.

**Table 1:** Summary of cases reported in HCC patients with colon/appendix metastasis.

Case	Year	Author	Age	Sex	Previous treatment	Time from initial HCC to colorectal/appendix metastasis (years)	History of tumor rupture	Site of metastasis	Treatment after finding of metastasis	Survival after the treatment
1	2021	Miyauchi et al. (20)	80	Male	Segment hepatectomy and radio-frequency ablation (RFA)	5.3	No	Ascending colon	Resection and lenvatinib	30 months
2	2021	Mu et al. (15)	86	Male	TACE and microwave ablation	10	No	Hepatic flexure	Colostomy	10 months
3	2020	Yu et al. (13)	60	Male	Hemihepatectomy and TACE	10	Yes	Descending colon	Hartmann Procedure	Over 3 months
4	2020	Kim et al. (18)	72	Male	Hemihepatectomy	3	No	Ascending colon	Resection	6 weeks
5	2019	Pham et al. (19)	60	Male	TACE	1	No	Sigmoid colon	Resection	Not mentioned
6	2019	Tagliabue et al. (14)	70	Male	TACE	No mentioned	No	Sigmoid colon	Resection	Not mentioned
7	2016	Wu et al. (21)	54	Male	Segment-hepatectomy	5	No	Ileocecal junction	Right-half colon resection	4 years
8	2010	Yoo et al. (16)	47	Male	TACE	1.5	No	Sigmoid colon	Anterior resection	Over 4 months
9	2008	Kim et al. (5)	50	Male	None	At the same time	Yes	Appendix	Appendectomy	Not mentioned

According to the Barcelona Clinic Liver Cancer (BCLC) staging system (17), the detection of extrahepatic metastasis indicates an advanced stage. Rather than resection, systemic treatment or conservative treatment would be recommended for such patients based on the BCLC staging system. The prognosis of such patients would be extremely poor, and the median survival time is expected to be nearly 1 year. However, according to the Chinese National Liver cancer stage system and the consensus on multidisciplinary management of recurrent and metastatic HCC after resection, resection could be recommended for patients with solitary metastasis (1, 7). The prognosis of recurrent HCC patients after repeat resection was found to be associated with the clinicopathologic characteristics of primary HCC and recurrence interval (7). In most reported cases mentioned in Table 1, surgery was performed to cure abdominal pain or intestinal obstruction rather than to cure HCC (13, 18–21). The overall survival since the detection of colon/appendix metastasis of the reported cases varied from 6 weeks to 5 years. The overall survival depends mainly on the liver function, tumor burden, and performance status of the patient. If the performance status is acceptable and complete resection is possible, aggressive resection might lead to a prolonged prognosis. For this patient, we found only recurrence in the tip of the appendix, which could be R0-resected at the first time, which means resection might be an acceptable choice.

Although R0 resection could be performed on HCC patients with solitary extrahepatic metastasis, a high risk of recurrence still existed (22). Because extrahepatic metastasis usually means that the tumor cell has penetrated into the blood vessel. Therapy used in patients with a high risk of recurrence was still under exploration. For now, only TACE has been confirmed with the effect of reducing the recurrent rate in random clinical trials (23). In this clinical trial, patients who received adjuvant TACE had a significantly longer 3-year recurrence free survival (RFS) than those who received conservative treatment (56% versus 42.1%). After 3 months, this patient returned to the department for adjuvant TACE. However, we found multiple intrahepatic recurrence in the routine examination. Recent advancements in tumor biology are currently attracting great interest in new antitumor drugs including ICIs and angiogenesis inhibitors (24). Given that the conventional locoregional therapies and angiogenesis inhibitors could induce the release of local inflammatory factors and neoantigens (25), several trials are assessing combination therapy for HCC patients without decompensation of liver function and surgical opportunity (26, 27). After combination therapy of conventional locoregional therapies, angiogenesis inhibitors, and ICIs, 33.3%–52.3% of unresectable patients could regain the opportunity of R0 resection. Among the reported cases, observative response rates are between 41.7% and 77.4%. With the inspiring results from recent clinical trials of TACE, immunotherapy, and target therapy (28), a combination of such treatment was suggested to this patient, and we received outstanding outcomes. After combining angiogenesis inhibitors, ICIs, and TACE, tumor markers came down to the normal range. In the routine examination, no sign of recurrence was detected.

For this patient, we did not perform a genetic test after we found tumor recurrence in the appendix or liver. Unlike other tumors, the genomic test is not necessary to treat advanced HCC. According to one investigation of phase III clinical trials (SHARP), angiogenesis markers [angiopoietin 2 (Ang2) and vascular endothelial growth factor (VEGF)] are predictors of overall survival in patients with HCC. However, neither Ang2 nor VEGF could predict a response to sorafenib (29). Lenvatinib, an oral inhibitor of vascular endothelial growth factor receptors (VEGFRs), fibroblast growth factor receptor 1-4 (FGFR1-4), ret proto-oncogene (RET), KIT proto-oncogene receptor tyrosine kinase, and Platelet-derived growth factor receptor α (PDGFRa), has been tested in phase III trials in advanced HCC (30). In this clinical trial, lenvatinib has been proven to be noninferior to sorafenib in terms of overall survival. Similarly, no biomarker-predicting responses to lenvatinib have been reported (31). ICIs, including agents targeting cytotoxic T lymphocyte protein 4, PD-1, or its ligand PD-L1, have been proven effective in many clinical trials (32, 33). Different from other tumors, data presented on nivolumab and pembrolizumab (PD-1 inhibitors) have not shown any correlation between PDL-1 expression or other biomarkers and treatment efficacy (31, 34). To date, in the angiogenesis inhibitors or ICIs used in HCC, no specific gene mutation has been confirmed that could stratify patients, and this may be the reason why no clinical guidelines for HCC highly recommended genetic test.

Overall, solitary metastasis to the appendix 5 years after R0 resection of the intrahepatic lesion is a really rare phenomenon in HCC. This case might be the only one reported so far. Because of its scarcity, no well-designed clinical trials could provide treatment suggestions for this situation. Appendectomy might be an acceptable choice for patients with solitary metastasis to the appendix. Metastasis of extrahepatic lesions usually means dissemination of tumor cells into the body and a high risk of recurrence. Combined therapy of local regional treatment, angiogenesis inhibitors, and ICIs might be used to deal with this situation even after R0 resection of metastatic lesions.

## Patient perspective

We contacted this patient in October 2022 and asked him for his views on our therapy. No complaints or questions were proposed. Before the surgery, this patient suffered from lower abdominal pain. After the surgery, no pain was found. With the following medical treatment, the tumor was controlled with complete response. He thought the treatment therapy was successful.

## Data Availability

The original contributions presented in the study are included in the article/Supplementary Material; further inquiries can be directed to the corresponding author.
